# Clinical Outcome and Safety of Transcaval Access for Transcatheter Aortic Valve Replacement as Compared to Other Alternative Approaches

**DOI:** 10.3389/fcvm.2021.731639

**Published:** 2021-09-22

**Authors:** Israel M. Barbash, Amit Segev, Anat Berkovitch, Paul Fefer, Elad Maor, Dan Elian, Ehud Regev, Victor Guetta

**Affiliations:** Interventional Cardiology Unit, Leviev Heart Center, Sheba Medical Center, Sackler Faculty of Medicine, Tel Aviv University, Ramat Gan, Israel

**Keywords:** transapical access, transcaval access, transaxillary access, transcatheter aortic valve replacement, aortic stenosis, arterial access

## Abstract

**Background:** A small proportion of patients in need of transcatheter aortic valve replacement (TAVR) are not suitable for the transfemoral approach due to peripheral artery disease. Alternative TAVR approaches are associated with short- and long-term hazards. A novel technique of caval-aortic (transcaval) access for TAVR has been utilized as an alternative access technique.

**Aim:** To compare safety and efficacy of transcaval access as compared to other alternative access (axillary or apical) for TAVR.

**Methods:** A single-center, retrospective analysis of consecutive patients undergoing alternative access for TAVR. Events were adjudicated according to VARC-2 criteria.

**Results:** A total of 185 patients were included in the present analysis. Mean age was 81 years with a small majority for male gender (54%). Of the entire cohort, 20 patients (12%) underwent transcaval TAVR, and 165 patients (82%) underwent TAVR using alternative access. Overall, baseline characteristics were comparable between the two groups. General anesthesia was not utilized in transcaval patients; however, it was routinely used in nearly all alternative access patients. TAVR device success was comparable between the two groups (95%). Acute kidney injury occurred significantly less frequently among transcaval patients as compared to alternative access patients (5 vs. 12%, *p* = 0.05). Hospital stay was shorter for transcaval patients (6.3 days vs. 14.4; *p* < 0.001). No difference in early or 30-day mortality (10 vs. 7.9%, *p* = 0.74) was noted between groups.

**Conclusions:** In patients who cannot undergo TAVR *via* the trans-femoral approach due to peripheral vascular disease, transcaval access is a safe approach as compared to other alternative access techniques, with lower risk of kidney injury and shorter hospital stay.

## Introduction

Transcatheter aortic valve replacement (TAVR) technology has transformed toward younger and lower-risk patients with severe aortic stenosis (1). This transformation has been facilitated by the availability of improved transcatheter heart valves and delivery platforms. However, at the other end of the spectrum there are still significant amount of patients who do not meet the anatomic criteria to undergo trans-femoral TAVR due to hostile ilio-femoral artery anatomy. These patients are typically sicker than the average TAVR patient and suffer from significant peripheral artery disease. While alternative access approaches for TAVR have been performed from the early days of this technology, worse outcomes have limited their use (2–4).

Development of the technique for caval-aortic (transcaval) access at the National Institute of Health allowed controlled traversal from the inferior vena cava into the infra-renal aorta using trans-catheter electrosurgery and, by that, bypassing diseased ilio-femoral arteries (5, 6). This technique has been utilized to perform TAVR in patients without suitable anatomy for trans-femoral approach.

While data to prove the safety and feasibility of transcaval access is accumulating both in prospective and retrospective registries, comparison to other alternative access techniques for TAVR is limited. Thus, the aim of the present study was to compare the procedural success and safety of transcaval TAVR vs. other alternative access TAVR, namely trans-apical and trans-axillary approaches.

## Methods

The study population included patients who underwent TAVR at Sheba Medical Center between February 2010 and April 2021. All subjects were referred to TAVR after careful evaluation by each institutional heart team. Default approach for TAVR during the entire study period was trans-femoral, while patients with unsuitable ilio-femoral anatomy for trans-femoral approach were referred for alternative access. Subjects undergoing trans-femoral TAVR were excluded from the present study.

Baseline data regarding past medical history and medications were recorded by a blinded investigator into a computerized database. Study data were prospectively collected and managed using REDCap electronic data capture tools hosted at The Israeli Center for Cardiovascular Research (7). Data were retrospectively analyzed for the purpose of the present study. All subjects underwent a detailed echocardiography before and after the procedure. Mortality data were ascertained by the Ministry of Internal Affairs Population Registry.

The institutional ethical review board approved the trial protocol, and the trial was conducted according to the principles of the Declaration of Helsinki. All patients provided signed informed consent to participate in the registry.

### Study Groups and Definitions

Subjects were divided into two groups based on access approach, namely, transcaval vs. other alternative access. History of ischemic heart disease, hypertension, diabetes mellitus, and stroke was extracted from patients' electronic medical history files based on known diagnoses or concurrent diabetic or blood pressure-lowering medications. Renal function was evaluated using the Modification of Diet in Renal Disease equation. Hospitalization course including use of inotropes, mechanical support, or need for intra-aortic balloon pump was documented. Periprocedural outcome and complications were recorded according to the Valve Academic Research Consortium-2 (8).

### Transcaval Access and Closure

**Figure 1:**
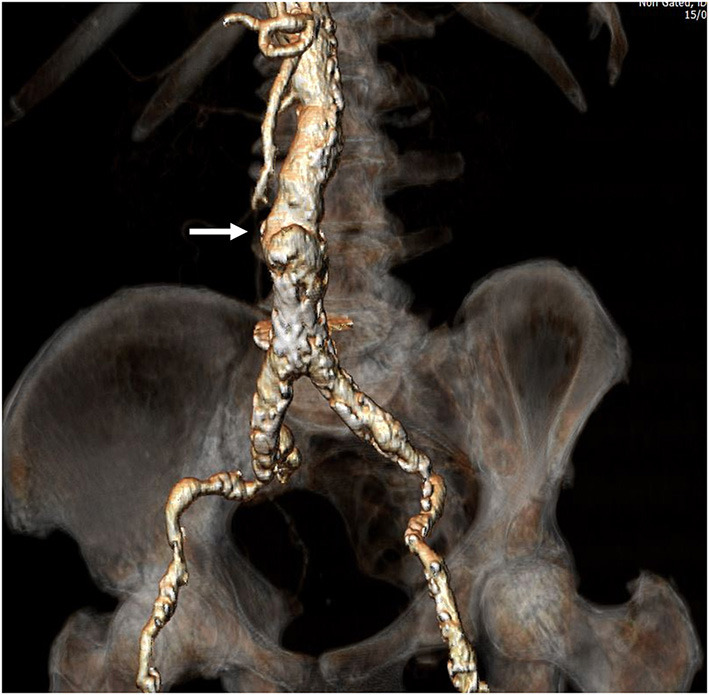
Preprocedural computed tomography angiography. Full evaluation of bilateral ilio-femoral arteries was performed assessing for minimal vessel diameter, extent and circumference of calcification, and vessel tortuosity. Once a patient was deemed as high-risk for standard trans-femoral access as shown in the figure, a calcium-free window in the infra-renal abdominal aorta was identified. This would be the target for traversal from the vena cava to the abdominal aorta (Arrow).

**Figure 2:**
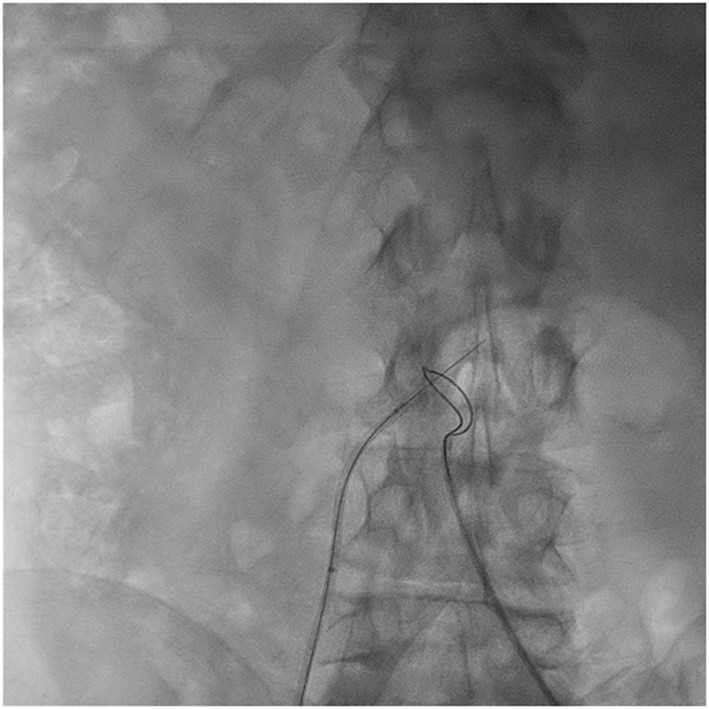
Caval to abdominal aorta traversal. A 0.014″ guidewire is positioned in the inferior vena cava pointing toward the abdominal aorta. A loop snare is positioned at the abdominal aorta at the level of the calcium-free window identified in preprocedural computed tomography. Using a catheter electrosurgery technique, the guidewire is electrified and advanced into the abdominal aorta to the center of the snare.

**Figure 3:**
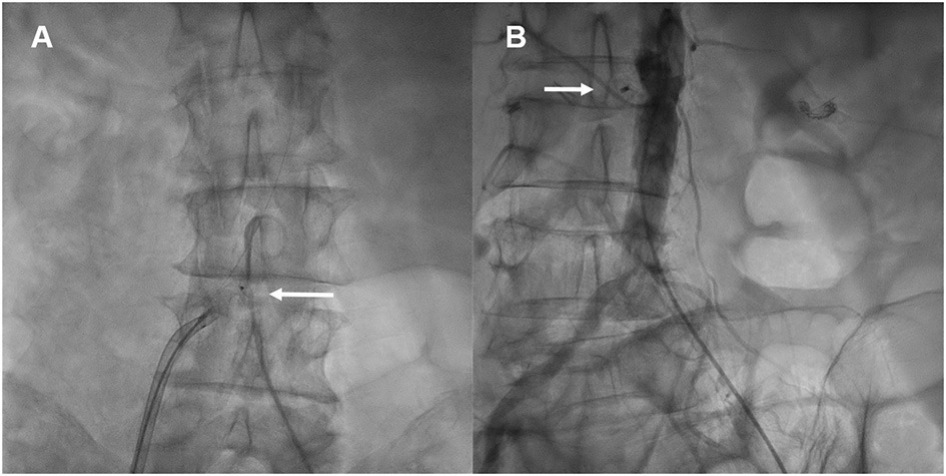
Hemostasis of abdominal aorta puncture. The nitinol occluder is deployed at the aorta puncture site using contrast injections from a pigtail catheter in the abdominal aorta **(A)**, and utilizing a deflectable sheath which allows for co-axial deployment of the occluder (**A**, Arrow). Once the occluder is positioned and released, a final aortogram is performed to verify successful hemostasis at the puncture site (**B**, Arrow).

Transcaval access was performed as previously described by Lederman and Greenbaum et al. (5, 9). Briefly, based on computed tomography angiography, a calcium-free window in the infra-renal abdominal aorta was identified and selected during pre-procedural evaluation (Figure 1). Utilizing an electrified 0.014″ guidewire (Astato XS 20 0.014 inch; Asahi Intecc Medical, Aichi, Japan), traversal from the inferior vena cava into the abdominal aorta is performed, where a snare (Amplatz Goose Neck Snare; Medtronic, Minneapolis, MN, USA) is positioned and snares the tip of the guidewire (Figure 2). Telescopic (mother-in-child) microcatheters (Finecross inside NaviCross; Terumo, Tokyo, Japan) are utilized to upgrade the traversal site and allow delivery of a 0.035 wire. Next, a stiff 0.035″ guidewire (Lunderquist Extra-Stiff Wire; Cook Medical, Bloomington, IN, USA) is delivered through the NaviCross catheter. Over the stiff guidewire, the procedural large bore sheath is delivered. Standard TAVR procedure is performed. Once the valve is deployed, the abdominal aortic access site is closed. This step is performed using a nitinol occluder (Abbott Cardiovascular, Plymouth, MN, USA) which seals the abdominal aorta wall (Figure 3). When incomplete hemostasis is achieved, sequential, prolonged, appropriately sized, peripheral balloon inflations are performed (AltoSa XL PTA Balloon; AndraTec Medical Devices, Koblenz, Germany), and if this measure fails to achieve hemostasis, a bail-out stent graft (BeGraft Peripheral Stent; Bentley, Hechingen, Germany) is used to obtain complete seal. Selection of transcatheter heart valve type was at the discretion of the treating physician and was based on anatomic considerations of the valve, sinus of Valsalva, coronary height, and presence of coronary artery disease.

### Tran-Apical and Trans-Axillary Procedures

Procedures were performed under general anesthesia. For trans-apical approach, cardiac surgeons performed mini-thoracotomy adjacent to the left ventricular apex; next, two purse string sutures were placed at the planned apical puncture site. Once apical puncture was performed, the interventional cardiology team performed balloon pre-dilatation of the aortic valve when needed and performed transcatheter valve implantation. Upon completion of valve implantation, the surgeon obtained hemostasis of the apical access site during rapid pacing of the ventricle. There was no routine use of a heart-lung machine or other circulatory assist device.

Trans-axillary access was obtained through a small infraclavicular incision in the deltopectoral groove. Arteriotomy and placement of a Goretex conduit were performed to provide access to the vessel. Delivery system insertion, balloon predilatation of the aortic valve, and valve implantation were performed by the interventional cardiology team. Arterial hemostasis was performed by the surgeon.

### Statistical Analysis

Continuous variables are described as mean and standard deviation or as median and interquartile range when not normally distributed. Normality was tested by the Kolmogorov–Smirnov test and Shapiro–Wilk tests. Categorical variables were compared using the chi-square or Fisher's exact test. Continuous variables with normal distribution were compared using independent-samples *t*-test. Continues variables with non-normal distribution were compared using Mann–Whitney *U*-test. Survival estimate was performed using the Kaplan–Meier and log-rank estimate.

Propensity-matched analysis was then performed using the nearest neighbor method comparing patients in the transcaval group to patients in the alternative access group. Parameters that were found to be significant in the univariate model or that are known to be significant in survival of patients undergoing TAVR were incorporated into the matching model. The matching included the following variables: age, gender, ischemic heart disease, diabetes mellitus, hypertension, previous stroke, and chronic kidney disease. Patients who underwent TAVR *via* the transcaval approach were matched to patients in the alternative access group, using individual propensity scores, in a 1:2 ratio.

Statistical significance was accepted for a two-sided *p* < 0.05. The statistical analyses were performed with IBM SPSS version 25.0 (SPSS, Chicago, IL, USA) and with SAS Enterprise Guide version 7.1 (SAS Institute Inc., Cary, NC, USA).

## Results

A total of 185 patients were included in the present analysis. Mean age of this cohort was 81 years with a small majority for male gender (54%). Coronary artery disease was prevalent in the majority of the patients (60%) and a third had a history of cardiac surgery. Other comorbidities were also frequent among the study cohort, including 72% chronic renal failure and 38% diabetes mellitus. Accordingly, median STS score was 4.6 and EuroSCORE II was 6 among the entire cohort (Table 1).

**Table 1:** Baseline characteristics of patients undergoing TAVR *via* transcaval vs. alternative access.

	**All patients**	**Transcaval access**	**Alternative access**	***p*-value**
	**(*n* = 185)**	**(*n* = 20)**	**(*n* = 165)**	
Age, Median (IQR)	82 (77, 86)	82 (80, 84)	83 (77, 86)	0.66
Males; *n* (%)	101 (55%)	15 (75%)	86 (52%)	0.052
History of coronary artery disease	111 (60%)	16 (80%)	95 (58%)	0.053
Prior myocardial infarction	39 (21%)	6 (30%)	33 (20%)	0.064
Prior percutaneous intervention	36 (20%)	11 (55%)	25 (15%)	<0.001
Prior coronary bypass surgery	57 (31%)	7 (35%)	50 (30%)	0.038
Prior stroke	32 (17%)	2 (10%)	30 (18%)	0.53
Diabetes	70 (38%)	10 (50%)	60 (36%)	0.4
Hypertension	156 (84%)	17 (85%)	139 (84%)	0.9
Chronic lung disease	27 (15%)	2 (10%)	25 (15%)	0.54
Atrial fibrillation	39 (21%)	7 (35%)	32 (19%)	0.25
Renal failure	134 (72%)	12 (60%)	122 (74%)	0.188
NYHA class III–IV	112 (61%)	18 (90%)	94 (57%)	0.022
STS score; Median (IQR)	4.6 (3.1, 6.3)	4.1 (2.9, 4.9)	4.7 (3.1, 6.5)	0.06
EuroSCORE II; Median (IQR)	6.0 (3, 10.5)	4.6 (2.6, 7.3)	7.0 (3.3, 11.7)	0.05
**Baseline echocardiography**				
Left ventricular ejection fraction; Median (IQR)	60 (45, 60)	53 (40, 60)	60 (47, 61)	0.17
Moderate/Severe mitral regurgitation	30 (16%)	5 (29%)	25 (17%)	0.22
Aortic valve area; Median (IQR)	0.70 (0.57, 0.80)	0.73 (0.57, 0.80)	0.7 (0.57, 0.8)	0.55
Peak aortic valve gradient; Median (IQR)	64 (52, 80)	64 (39, 78)	64 (53, 80)	0.23
Mean aortic valve gradient; Median (IQR)	40 (31, 51)	40 (24, 51)	40 (31, 51)	0.28

*IQR, interquartile range*.

Of the entire cohort, 20 patients (12%) underwent transcaval TAVR, and 165 patients (82%) underwent TAVR using alternative access (either apical or axillary). Overall baseline characteristics were comparable between the two groups (Table 1). However, there was a tendency toward male predominance (75 vs. 52%; *p* = 0.052) and history of coronary artery disease (80 vs. 58%; *p* = 0.053) in the transcaval vs. the alternative access group. Both STS score (4.4 vs. 5.8%; *p* = 0.06) and EuroSCORE 2 (6.1 vs. 9.2%; *p* = 0.05) tended to be lower in the transcaval group. There were no differences between groups with regard to left ventricular function, aortic stenosis severity, or concomitant mitral regurgitation.

General anesthesia was not utilized in transcaval patients; however, it was routinely used in nearly all alternative access patients (Table 2). There was strong predominance of self-expandable valve utilization among transcaval patients, while there was a strong predominance of balloon expandable valves in the alternative access patients. Device success according to VARC-2 criteria was high in both groups 95%, while the driver for device failure in both groups was the use of a second transcatheter valve.

**Table 2:** Procedural characteristics and outcome of patients undergoing TAVR *via* the transcaval vs. alternative access.

	**Transcaval**	**Alternative**	***p*-value**
	**access**	**access**	
	**(*n* = 20)**	**(*n* = 165)**	
**Procedural data**			
General anesthesia	0 (0%)	164 (99%)	<0.001
Valve type			<0.001
Self-expandable	15 (75%)	39 (25%)	
Balloon expandable	5 (25%)	125 (75%)	
Device success	19 (95%)	156 (95%)	0.79
**Procedural complications**			
Conversion to surgery	0 (0%)	6 (3.6%)	0.46
Coronary obstruction	0 (0%)	1 (0.6%)	0.64
Tamponade	0 (0%)	3 (1.8%)	0.56
Annular rupture	0 (0%)	1 (0.6%)	0.64
Need for a second valve	1 (5%)	8 (4.8%)	0.69
**In-hospital complications**			
Periprocedural myocardial infarction	0 (0%)	3 (1.8%)	0.3
Stroke	2 (10%)	7 (4.2%)	0.23
Life-threatening or major bleeding	2 (10%)	25 (15%)	0.55
Acute kidney injury (Grade 2–3)	1 (5%)	20 (12%)	0.05
Acute hemodialysis	0 (0%)	7 (4.2%)	0.36
Major vascular complication	0 (0%)	7 (4.2%)	0.13
Permanent pacemaker	3 (15%)	22 (13%)	0.56
In-hospital mortality	1 (5%)	17 (10.3%)	0.45
Length of hospitalization	6 (4, 7)	8 (5, 16)	<0.001
Discharge home	13 (65%)	88 (53%)	0.73
30-day mortality	2 (10%)	13 (7.9%)	0.74

As shown in Table 2, acute kidney injury (levels 2 or 3) occurred significantly less frequently among transcaval patients as compared to alternative access patients (5 vs. 12%, p=0.05) with a non-significant difference in the need for acute dialysis (0 vs. 4.2%; *p* = 0.36), respectively. Apart from kidney injury, incidence of other in-hospital periprocedural complications did not show any statistically significant differences between the two groups. Stroke or TIA rates were comparable between the transcaval vs. alternative access groups (10 vs. 4.2%; *p* = 0.2) as well as life-threatening or major bleeding (10 vs. 15%; *p* = 0.55) and major vascular complications (0 vs. 4.2%; *p* = 0.13), respectively. In the transcaval group, one aortic stent graft was used due to failure to deploy a nitinol plug with no clinical consequences. In-hospital mortality was comparable between the groups (5 vs. 10.3%, *p* = 0.45).

**Figure 4:**
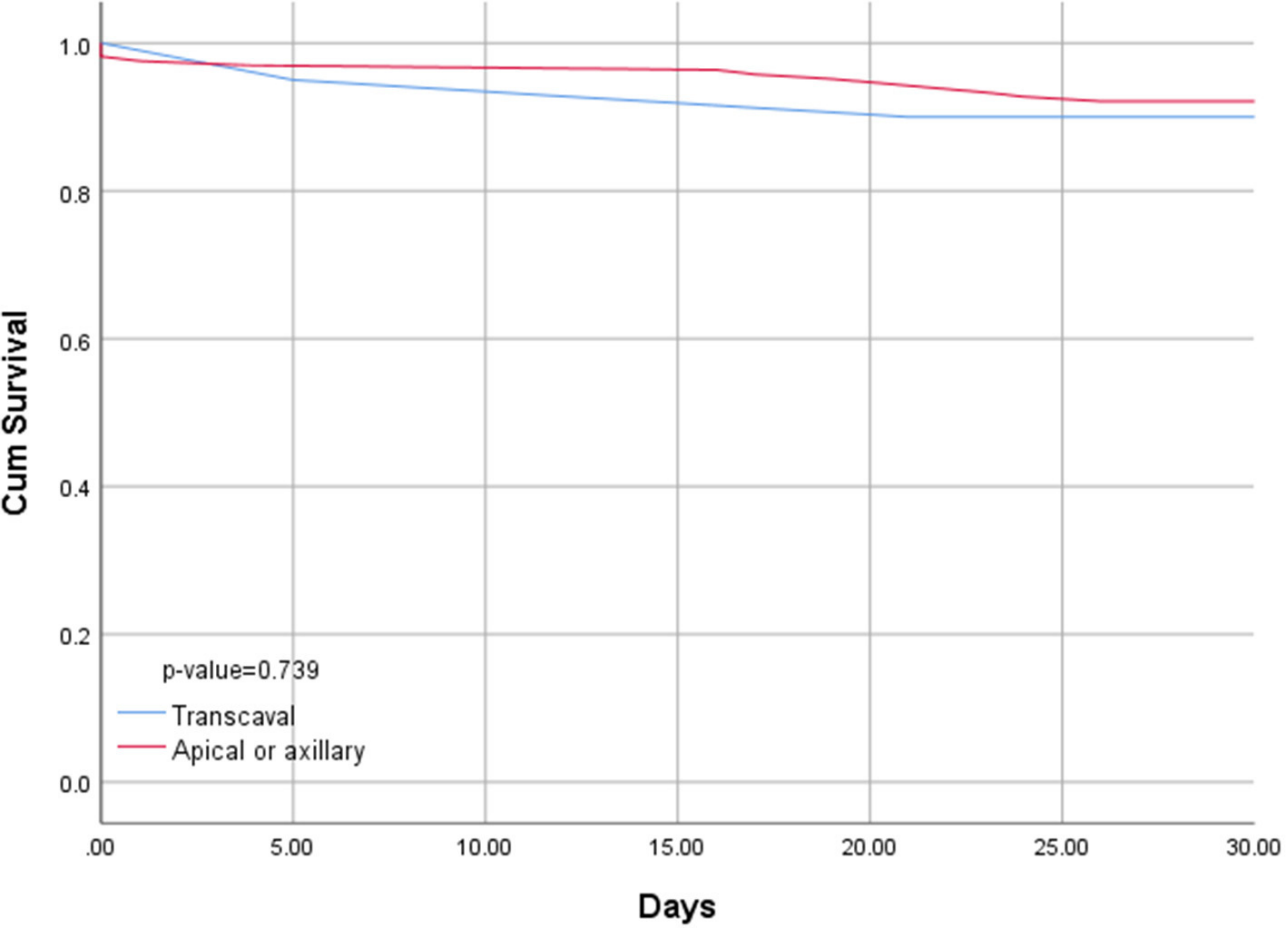
Kaplan–Meyer survival curve of patients undergoing transcaval vs. alternative access transcatheter aortic valve replacement.

Length of hospital stay for transcaval patients was significantly shorter than the length of stay for alternative access patients (6.3 days vs. 14.4; *p* < 0.001). Transcaval patients were discharged directly to their homes without the need for rehabilitation; more frequently (65 vs. 53%), however, this difference did not reach statistical significance. Thirty-day mortality was comparable between the two groups (10 vs. 7.9%, respectively, *p* = 0.74) (Figure 4).

Propensity score matching of transcaval vs. alternative access groups (Supplementary Table 1) showed that use of self-expandable valves was more frequent in the transcaval group. In terms of procedural and in-hospital outcomes (Table 3), findings were comparable to the findings in the entire cohort. Procedural complication rates were comparable between the two groups. In-hospital outcomes were overall comparable; however, the rates of acute kidney injury were significantly lower, and hospitalization length was significantly shorter in the transcaval group as compared to the alternative access group (Table 3).

**Table 3:** Propensity score matched procedural characteristics and outcome of patients undergoing TAVR *via* the transcaval vs. alternative access.

	**Transcaval**	**Alternative**	***p*-value**
	**access**	**access**	
	**(*n* = 20)**	**(*n* = 40)**	
**Procedural data**			
General anesthesia	0 (0%)	40 (100%)	<0.001
Valve type			<0.001
Self-expandable	15 (75%)	12 (30%)	
Balloon expandable	5 (25%)	28 (70%)	
Device success	19 (95%)	39 (100%)	0.79
**Procedural complications**			
Conversion to surgery	0 (0%)	2 (5%)	0.31
Coronary obstruction	0 (0%)	0 (0%)	1.00
Tamponade	0 (0%)	1 (2.5%)	0.47
Annular rupture	0 (0%)	0 (0%)	1.00
Need for a second valve	1 (5%)	2 (5%)	1.00
**In-hospital complications**			
Periprocedural myocardial infarction	0 (0%)	1 (2.5%)	0.47
Stroke	2 (10%)	3 (7.5%)	0.38
Life-threatening or major bleeding	2 (10%)	4 (10%)	0.53
Acute kidney injury (Grade 2–3)	1 (5%)	6 (15%)	0.08
Acute hemodialysis	0 (0%)	2 (5%)	0.28
Major vascular complication	0 (0%)	0 (0%)	1.00
Permanent pacemaker	3 (15%)	4 (10%)	0.41
In-hospital mortality	1 (5%)	6 (16.2%)	0.53
Length of hospitalization, median (IQR)	6 (4, 7)	7 (5, 14)	0.008
Discharge home	13 (65%)	23 (57%)	0.75
30-day mortality	2 (10%)	2 (5%)	0.46

## Discussion

The present study addresses the important issue of TAVR access site considerations among patients who are not suitable for standard trans-femoral access. The main findings of the present analysis indicate that among patients who are not suitable for the trans-femoral approach, the transcaval approach for TAVR is safe and effective at least as other commonly used alternative access approaches (trans-axillary and trans-apical approaches). Furthermore, the transcaval approach did not require general anesthesia and was associated with lower rates of acute kidney injury and shorter hospital stay.

Data regarding the safety of alternative access routes for TAVR is inconclusive. Large-scale analysis from the Society of Thoracic Surgeons/American College of Cardiology (STS/ACC) Transcatheter Valve Therapies Registry (10) has indicated that non-trans-femoral access for TAVR is associated with increased risk for 1-year mortality. Several studies have shown that the trans-apical approach is associated with significant increase in mortality and with trends for higher complication rates of stroke, cardiac tamponade, pacemaker implantation, and longer hospitalization (2, 3). Conversely, other studies have suggested comparable outcomes between the two approaches and associated the outcome differences to the sicker patient profile of the trans-apical patients and to operator experience in the technique (11, 12).

A similar controversy exists with regard to other alternative access techniques such as the trans-axillary approach which shares the same long-term hazards as the trans-apical approach (4). Given these concerns of the increased short- and long-term risk of the sick patient population with peripheral vascular disease, other novel methods to perform TAVR with a minimally invasive approach have been developed over the last few years.

Aortic access from the vena cava (transcaval access) was initially developed at the National Institute of Health (6). This approach uses trans-catheter electrosurgery principles (13) in order to traverse with a wire from the vena cava to the infra-renal abdominal aorta and was initially developed to overcome hostile, diseased ilio-femoral arteries, which will not allow the delivery of large bore devices and sheaths. Upon completion of the procedure, the large bore aortic access is closed with a nitinol occluder (Abbott Cardiovascular). The physiological basis behind the transcaval approach is based on pressure gradients between three separate chambers, namely, the vena cava with the lowest pressure, aorta with the highest pressure, and the retroperitoneum within which both the vena-cava and aorta lie, with a pressure higher than the vena cava but lower than the aorta. Accordingly, if bleeding from the aorta occurs, the blood will follow to the lowest pressure chamber, i.e., the vena cava leading to the aortic-venous fistula, but not frank retroperitoneal bleeding, thus allowing the operator the ability to obtain hemostasis while the patient is hemodynamically stable.

Greenbaum et al. reported the results of the transcaval approach for TAVR among 100 consecutive patients enrolled in a prospective registry (14). In this analysis, transcaval access and closure were successful in 99% of the cases, with no procedural mortality or a need for urgent vascular surgery. In-hospital complications were comparable to the present analysis with low rates of kidney injury, stroke, and bleeding. A small number of aortic stent grafts were used in this study as opposed to one case in the present study, probably related to early experience with this technique. Finally, a 30-day mandatory computed tomography demonstrated that the aorto-caval fistula was occluded in the majority of the patients (72%). The findings of the present analysis are in agreement with these prior reports. Two smaller studies have compared transcaval access with alternative access (15, 16). First, a study by Paone et al. compared the transcaval approach in 58 patients to the standard trans-femoral approach and trans-carotid approach showing that short- and long-term outcome is comparable among the three approaches despite the fact that transcaval patients represented a sicker patient population (16). A more recent study by Long et al. compared the transcaval approach in 22 patients to the subclavian approach. In this study, a signal of higher early stroke rates and pacemaker were found in the subclavian access group, however without any difference in mortality (15). Currently, there are no data to compare other TAVR approaches, such as direct aortic, to the transcaval access.

The findings from the present analysis as well as prior reports on outcome after transcaval procedures support performing the transcaval approach as a default strategy if a patient has hostile ilio-femoral anatomy, in centers that are familiar with the transcaval access and closure. Other alternative access approaches should probably be considered in patients who have severe calcification of the abdominal aorta, which may preclude transcaval access.

## Limitations

The present study has several limitations. First, this is a retrospectively analyzed prospective registry, which is a non-randomized, non-blinded observational study, and therefore it is subjected to limitations inherent in this design. Second, this analysis represents data from a single center, and third, given the small groups and low event rates, no multivariable adjustments could have been performed to assess differences between groups.

## Conclusions

In patients who cannot undergo TAVR *via* the trans-femoral approach due to peripheral vascular disease, transcaval access is a safe approach as compared to other alternative access techniques, with lower risk of kidney injury and shorter hospital stay.

## Data Availability Statement

The raw data supporting the conclusions of this article will be made available by the authors, without undue reservation.

## Ethics Statement

The studies involving human participants were reviewed and approved by Sheba Medical Center. The patients/participants provided their written informed consent to participate in this study.

## Author Contributions

IB, AB, AS, PF, EM, ER, VG, and DE: conceive and perform procedures, study design and data collection, and manuscript writing and editing. IB, VG, and AB: statistical analysis. All authors contributed to the article and approved the submitted version.

## Conflict of Interest

The authors declare that the research was conducted in the absence of any commercial or financial relationships that could be construed as a potential conflict of interest.

## Publisher's Note

All claims expressed in this article are solely those of the authors and do not necessarily represent those of their affiliated organizations, or those of the publisher, the editors and the reviewers. Any product that may be evaluated in this article, or claim that may be made by its manufacturer, is not guaranteed or endorsed by the publisher.
