# Supplementary material for: Clinical Outcome and Safety of Transcaval Access for Transcatheter Aortic Valve Replacement as Compared to Other Alternative Approaches
Source: Front Cardiovasc Med. 2021 Sep 22;8:731639. doi: 10.3389/fcvm.2021.731639 (PMC8492973; doi:10.3389/fcvm.2021.731639)
Supplement: Supplementary file 1 [file Table_1.DOCX]

**Online Table 1: Propensity score matched baseline characteristics of patients undergoing TAVR via the transcaval versus alternative access**

|  | Transcaval access (n=20) | Alternative access (n=40) | P value |
| --- | --- | --- | --- |
| Age, median (IQR) | 82 (80, 84) | 83 (76, 85) | 0.38 |
| Males; n (%) | 15 (75%) | 27 (67%) | 0.55 |
| History of coronary artery disease | 16 (80%) | 33 (82%) | 0.81 |
| Prior myocardial infarction | 6 (30%) | 12 (32%) | 0.67 |
| Prior percutaneous intervention | 11 (69%) | 7 (19%) | <0.001 |
| Prior coronary bypass surgery | 7 (44%) | 21 (54%) | 0.49 |
| Prior stroke | 2 (10%) | 8 (20%) | 0.33 |
| Diabetes mellitus | 10 (50%) | 24 (60%) | 0.46 |
| Hypertension | 17 (85%) | 30 (75%) | 0.37 |
| Chronic lung disease | 2 (10%) | 6 (15%) | 0.59 |
| Atrial fibrillation | 7 (35%) | 7 (17%) | 0.13 |
| Renal failure | 12 (60%) | 26 (65%) | 0.70 |
| NYHA class III-IV | 18 (90%) | 26 (70%) | 0.09 |
| STS score (IQR) | 4.1 (2.9, 4.9) | 4.9 (3.2, 6.0) | 0.30 |
| EuroSCORE II (IQR) | 4.6 (2.6, 7.4) | 8.3 (3.4, 13.9) | 0.04 |
| **Baseline echocardiography** |  |  |  |
| Left ventricular ejection fraction, median (IQR) | 53 (40, 60) | 60 (55-60) | 0.16 |
| Moderate/Severe mitral regurgitation | 5 (29%) | 8 (22%) | 0.57 |
| Aortic valve area, median (IQR) | 0.73 (0.57, 0.8) | 0.71 (0.6-0.8) | 0.34 |
| Peak aortic valve gradient, median (IQR) | 64 (39, 78) | 64 (53, 80) | 0.38 |
| Mean aortic valve gradient, median (IQR) | 40 (24, 51) | 40 (32, 52) | 0.40 |
